# Supplementary material for: Evaluation of Polymer-Coated Carbon Nanotube Flexible Microelectrodes for Biomedical Applications
Source: Bioengineering (Basel). 2023 May 26;10(6):647. doi: 10.3390/bioengineering10060647 (PMC10295676; doi:10.3390/bioengineering10060647)
Supplement: Supplementary file 1 [file bioengineering-10-00647-s001.zip › bioengineering-2378966-supplementary.pdf]

Supplementary Information

Evaluation of Polymer-Coated Carbon Nanotube Flexible Microelectrodes for Biomedical Applications

†Chethani K. Ruhunage, ‡Vaishnavi Dhawan, †Chaminda P. Nawarathne, †Abdul Hoque, ‡*Tracy Cui , †*Noe T. Alvarez

†Department of Chemistry, University of Cincinnati, Cincinnati, OH 45221, USA
‡Department of Bioengineering, University of Pittsburgh, Pittsburgh, PA 15261, USA
*Corresponding Author: alvarene@ucmail.uc.edu
xic11@pitt.edu

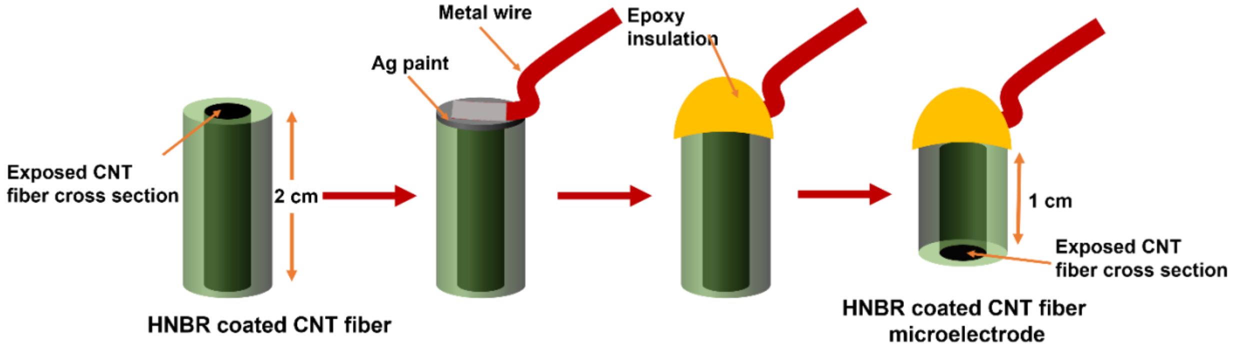

Figure S1: Schematic illustration of HNBR coated CNT fiber microelectrode fabrication.

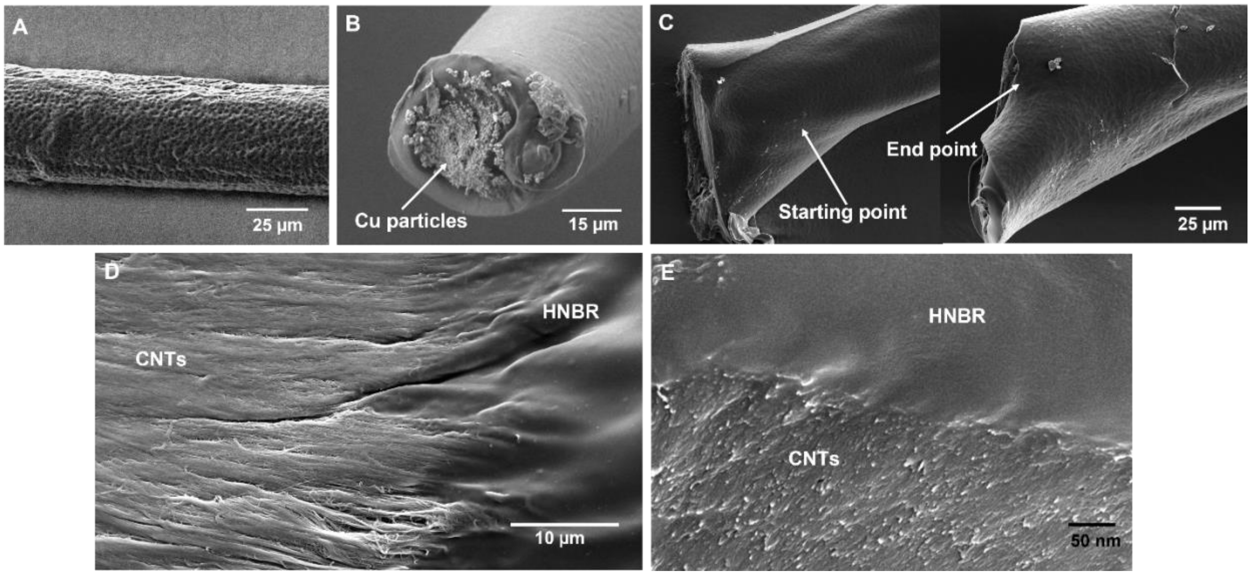

Figure S2: (A) SEM image of a 44 µm CNT fiber after coating by CDC with a withdrawal speed of 6 mm s^{-1} in a 0.055 g mL^{-1} HNBR dispersed solution. (B) SEM image of a HNBR coated CNT fiber microelectrode after electrodeposition of Cu using chronoamperometry at -0.2 V for 10 s in 50 mM $CuSO_4$. (C) SEM image of

the opposite ends of the 4.8 m long HNBR coated CNT fiber. (D) SEM image of HNBR polymer film forming at the meniscus of CDC process, and (E) A high magnification SEM image of the HNBR coated CNT fiber cross section revealing the seamless integration of the polymer with the CNT fiber at the interface.

In neural implants, complete insulation of underlying conductive elements is critical for reliable and safe use of the neural electrodes. Neural stimulating electrodes with coating defects could lead to undesirable large electrical current discharge into neural tissues. Also, defects free insulating coatings are extremely important for neural recording electrodes and biosensors to obtain stable and accurate information overtime. The investigation of defects and coating irregularities is critical for novel polymer coating materials and coating strategies. The possible defects and insulation were analyzed for CNT fibers coated under optimized conditions. Electrodeposition of Cu was used to detect coating defects and irregularities. Figure 4B exhibits a SEM image of the HNBR polymer coated CNT fiber microelectrode after performing electrodeposition of Cu. The microelectrodes were fabricated as mentioned in section 2.6 and 24.8 µm diameter CNT fiber with 7 µm thick coating was evaluated and reported here. A small portion of the microelectrode was shown in Figure 4B. The microelectrode was electrochemically pretreated before performing electrodeposition of Cu. The SEM image of sidewalls and the microelectrode area of a HNBR coated CNT fiber microelectrode after Cu deposition is shown. Cu particles were only found on CNT fiber cross section microelectrode area where the exposed CNTs directly interact with the Cu solution while No Cu deposits were found on the fiber sidewalls convincing no pinholes were formed by CDC process. Also, it further confirmed that 7 µm HNBR polymer thickness is sufficient for complete insulation of the CNT fiber.

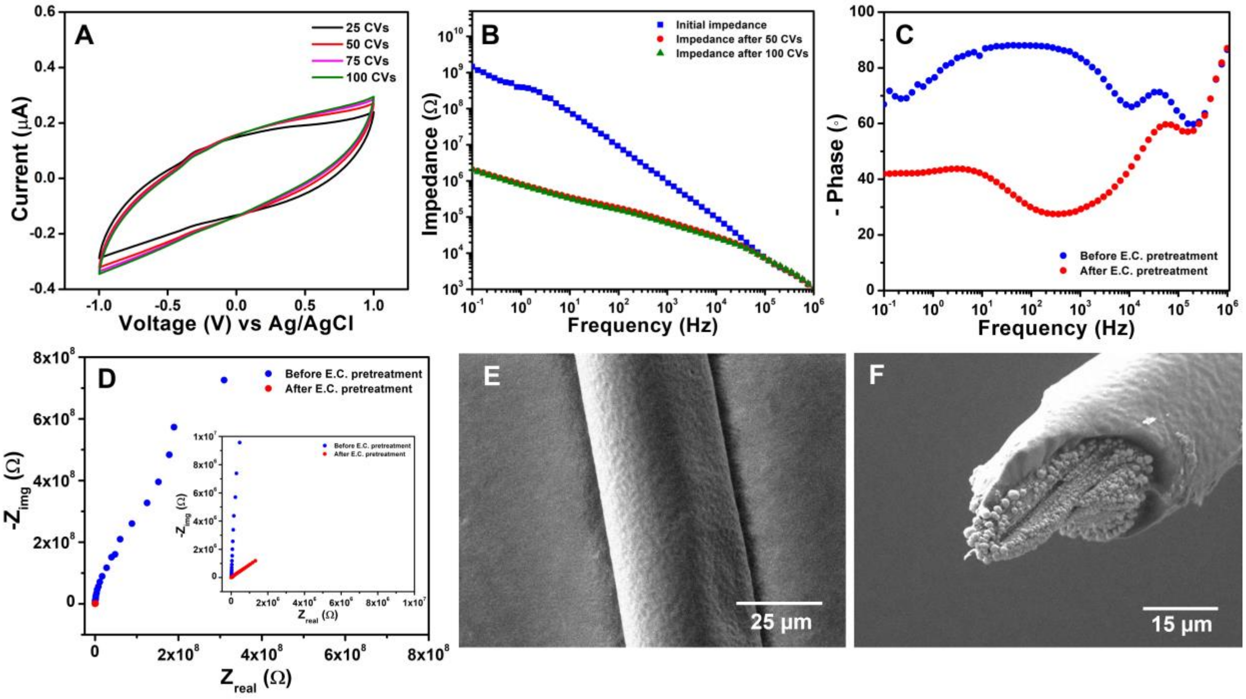

Figure S3: (A) Electrochemical pretreatment of a HNBR coated CNT fiber microelectrode by repetitive application of CVs in 0.01 M PBS with a scan rate of 0.1 V s^{-1}, (B) Impedance analysis of the microelectrode after 50 CVs (red) and 100 CVs (blue) are shown (Initial impedance was shown in black), (C) Negative phase angle as a function of frequency of a HNBR coated CNT fiber microelectrode before (blue) and after 50

CVs (red) in 0.01 M PBS, (D) Nyquist plot of the HNBR coated CNT fiber microelectrode shown in S3C, (E) SEM image of the side view of a portion of the HNBR coated CNT fiber microelectrode subjected to 200 repetitive CVs after chronoamperometry at -0.2 V for 10 secs in 50 mM $CuSO_4$, (F) SEM image of the Cu deposited active electrode area of the microelectrode shown in S3E.

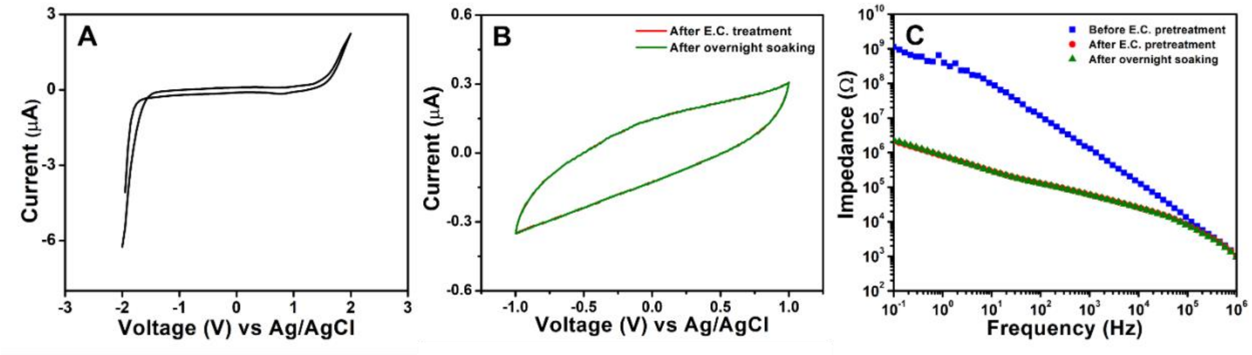

Figure S4: (A) The water window of HNBR coated CNT fiber microelectrode. (B) CV of a HNBR coated CNT fiber microelectrode after overnight soaking in 0.01 M PBS (red and green overlapping). CV was performed in 0.01 M PBS (pH 7.4) with a scan rate of 0.1 V s^{-1}. (C) EIS of a HNBR coated CNT fiber microelectrode after overnight soaking in 0.01 M PBS.

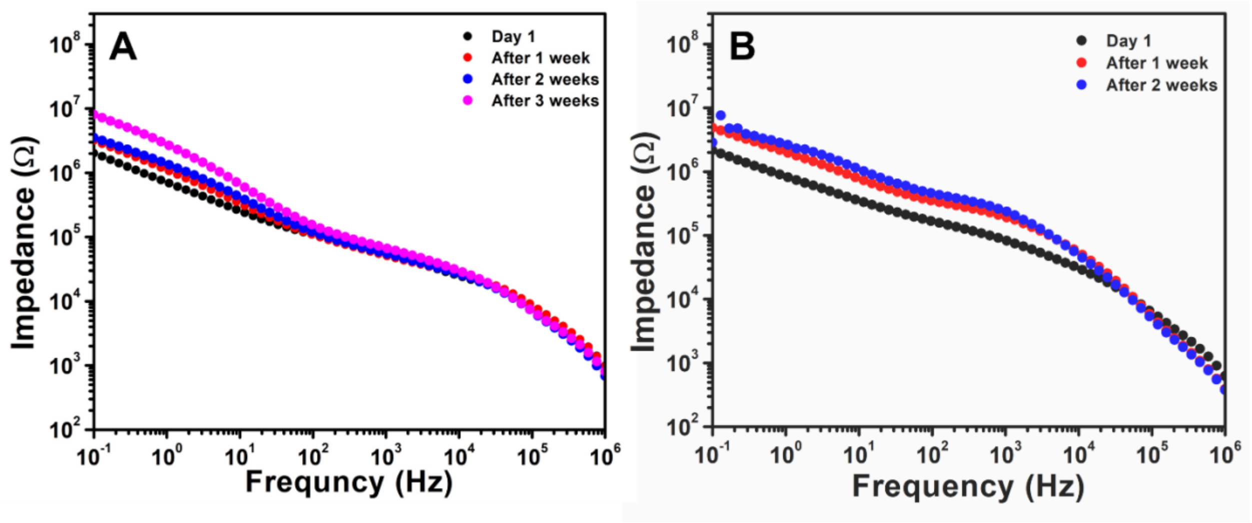

Figure S5: The Bode plot of a HNBR coated CNT fiber microelectrode (A) incubated at 37 °C for 3 weeks in PBS, and (B) incubated at 60 °C for 2 weeks in PBS. The EIS was performed in the frequency range of 0.1 to 10^6 Hz in 0.01 M PBS.
